# DNA signatures for detecting genetic engineering in bacteria

**DOI:** 10.1186/gb-2008-9-3-r56

**Published:** 2008-03-18

**Authors:** Jonathan E Allen, Shea N Gardner, Tom R Slezak

**Affiliations:** 1 Lawrence Livermore National Lab, Livermore, CA 94550, USA.

## Abstract

Using newly designed computational tools we show that, despite substantial shared sequences between natural plasmids and artificial vector sequences, a robust set of DNA oligomers can be identified that can differentiate artificial vector sequences from all available background viral and bacterial genomes and natural plasmids. We predict that these tools can achieve very high sensitivity and specificity rates for detecting new unsequenced vectors in microarray-based bioassays. Such DNA signatures could be important in detecting genetically engineered bacteria in environmental samples.

## Abstract 2

New computational tools were used to find a robust set of DNA oligomers that can distinguish artificial vector sequences from all available background viral and bacterial genomes.

## Background

Synthetic vector sequences are of fundamental importance in molecular biology. Cloning and expression vectors are among a multitude of synthetic sequence types commonly used as part of a basic tool set for DNA amplification and protein production [1]. As the emerging maturity of synthetic biology research fast approaches [2], it is reasonable to imagine in the not too distant future the broad-scale manufacture of sophisticated synthetic plasmids to modify existing bacteria and possibly the construction of new functioning synthetic genomes [3]. The potential exists to address challenges in many areas, from food production [4] to drug discovery [5]. However, along with the potential benefit comes the increased risk of engineered pathogens [6,7]. Thus, with improvements in genetic manipulation comes the need for tools to detect genetically modified bacteria in the environment.

Large-scale computational pipelines have advanced bio-defense by efficiently finding polymerase chain reaction (PCR) assay-based primers that are able to accurately identify dangerous bacterial and viral pathogens [8-10]. The development of random DNA amplification methods have highlighted microarrays as a potentially practical multiplexing complement to PCR [11] with DNA signatures on microarrays [12]. Recent progress has made DNA signature design tools widely available to pathogen research through the development of a publicly available computational pipeline for designing PCR-based signatures [13]. These advances demonstrate the utility of DNA signature pipelines, but the question remains whether such an approach could be used to detect genetically engineered bacteria.

A computational analysis was performed on the available synthetic vector sequences, which form an important basis for current tools in genetic engineering [14]. One of the results of this work is a report on the presence of DNA signatures found to differentiate the vector sequences from the sequenced naturally occurring plasmid and chromosomal DNA. Candidate DNA signatures were found to cover nearly all artificial vector sequences using a wide range of signature lengths. The presence of these candidate DNA signatures opens the potential to develop assays in the future for detecting simple but widely available forms of genetic engineering. The vector sequence data was further leveraged to predict natural plasmids, which may form the basis for future vectors based on conserved functional sequences.

## Results and discussion

### Vector DNA signatures

A total of 3,799 partial and complete artificial vector sequences totaling 21,132,057 nucleotides were collected from various sequence databases (details given in Materials and methods) and analyzed for conserved sequence elements. Sequences were compared using exact *k*-mer matching (a *k*-mer is a nucleic acid sequence of length *k*). This alignment-free comparative sequence approach [15,16] contrasts with methods that use conserved order among compared sequences [17]. The alignment-free comparison is motivated by the abundance of similar artificial vector sequences, which can differ in the relative order of functional elements owing to differing sources of sequence construction. Conserved order comparison is further confounded by transposable elements and the need to efficiently compare several thousand sequences simultaneously.

**Figure 1:**
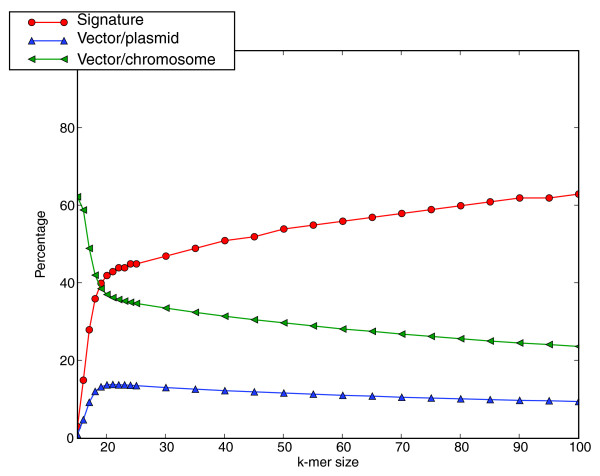
Percentage of *k*-mers that are candidate signatures. The red line plots the percentage of candidate vector signatures as a function of *k *(100% for a given *k *would mean all observed *k*-mers are signatures). The blue and green lines plot the percentage of artificial vector derived *k*-mers shared exclusively with natural plasmids and chromosomes, respectively.

A *k*-mer found in the vector sequence but not in the natural plasmid or chromosomal DNA is a candidate signature. The length of *k *was varied to examine the change in candidate signature set size; the results are shown in Figure 1 (red line with circles). There is a large jump in the percentage of *k*-mers that are candidate signatures going from 15 to 18 with a continued gradual increase as *k *increases above 18. The other lines in Figure 1 show the percentage of vector *k*-mers shared exclusively with the natural plasmid sequence (blue triangles) and chromosome sequence (green triangles). More vector derived 15-mers are shared with the chromosome sequence (62%) than with the natural plasmid sequence (1%) which is not surprising since there are over 4 billion bases of background viral and microbial sequence and less than 66 million bases of sequenced natural plasmids. Nevertheless, the gap narrows considerably at *k *= 18 with the chromosomal sequence showing a much smaller percentage of *k*-mer matches, suggesting that many of the matches under 18 are a result of random chance.

*k*-mer sets collapse the redundant candidate signatures. A *k*-mer set *X *for sequences from a set of input sequenced vectors *Y *is the set of *k*-mers shared by all *n *sequences where *n *is maximal. (There can be no additional input vector sequence in *Y *with the same set of shared *k*-mers not included in *X*.) For example, with three sequences *S*_1_, *S*_2 _and *S*_3_, if *S*_1 _and *S*_2 _share 20 *k*-mers not found in *S*_3_, these 20 *k*-mers would form a single *k*-mer set with a pointer to the two source sequences *S*_1 _and *S*_2_. If additional *k*-mers are shared with all three sequences *S*_1_, *S*_2 _and *S*_3_, these *k*-mers would form a separate *k*-mer set with a pointer to all three sequences.

**Figure 2:**
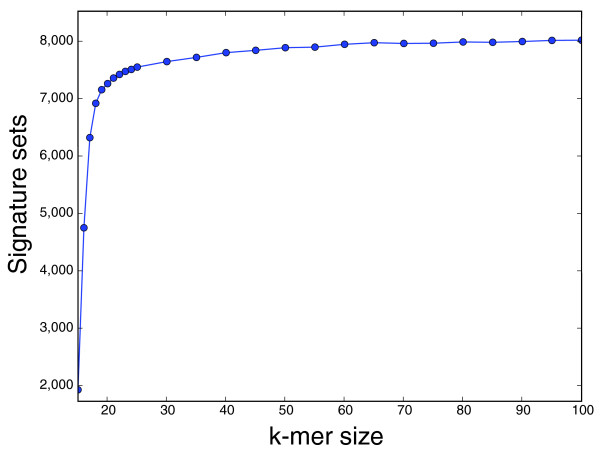
Signature sets. Plots of the number of *k*-mer sets containing signatures for *k *= 15 to 100.

A candidate signature set is a *k*-mer set where *k*-mers in the set are found in the vector data but not in the natural plasmid or chromosomal DNA. Using *k *= 20 as an example, the 1,625,171 signature candidates reduce to 7,270 signature sets, each with at least 10 signatures from which representative signatures can be chosen. Intuitively, shorter *k*-mers should reduce the number of candidate signatures, but Figure 2 shows that the signature set size levels off at *k *= 50. This means that longer signatures can be easily managed without creating a signature candidate pool that is too large. The candidate signature set size is reduced further using a greedy algorithm to iteratively select the *k*-mer set that maximally increases the number of sequences covered, reducing the size to 364 (when *k *= 20).

**Figure 3:**
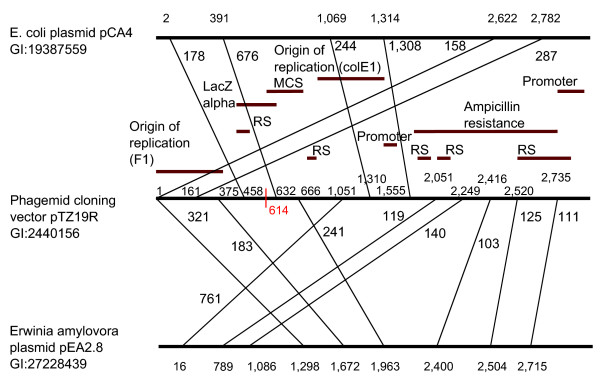
Example artificial vector sequence mapped to two natural plasmids. The vector sequence is shown in the middle (Phagemid cloning vector pTZ19R), which shares sequence with both the *E. coli *plasmid pCA4, and the *Erwinia amylovora *plasmid pEA2.8. Lines connecting the three sequences mark the beginning of exact matches between the artificial sequence and the two respective plasmids. The number next to each line is the length of exact match (for matches of 100 or more bases). Functional annotation for the artificial vector sequence is given above the sequence (RS denotes recombination site). Position 614 marks the starting point of the shortest signature found (*k *= 23). (Not drawn to scale.)

Eleven complete sequence vectors were found to be without a unique signature up to *k *= 47. For 9 of the 11 cases, the vector sequence and the natural sequence are identical. At *k *= 23 and 47, a signature is found for the remaining two sequences. Figure 3 shows a schematic of the overlap between the artificial vector sequence where the first signature appears at *k *= 23 and the natural plasmids with the two highest numbers of shared nucleotides. (Note that, for clarity, matches to other natural plasmid sequences are not shown.) The figure shows maximal exact matches over 100 bases in length using MUMmer [18]. We found that 99.6% of the vector sequence maps to the *Escherichia coli *plasmid with exact matches and 86% matches exactly to the *Erwinia amylovora *plasmid. A signature first emerges at the multiple cloning site at position 614 (shown in Figure 3). Overall, the choice of *k *yields only moderate changes in the signature set size and coverage. If microarrays are used as the assay medium, the choice of probe lengths can be tailored to fit a particular microarray design [19].

The completely sequenced vectors were divided into five partitions to check how closely vectors excluded from the signature creation pipeline match the candidate signatures. The hope is that a high percentage of the signatures are found in unseen vectors while remaining distinct from the background genomic sequence. The background genomic sequence is defined here as all sequenced natural plasmids and all sequenced bacterial and viral chromosomes along with the assembled draft sequence. Each partition was searched against a signature set generated from the remaining 80% of the vector data using NCBI BLAST [20]. The background genomic sequence was similarly searched against each of the five signature sets. Each vector sequence and background genomic sequence was assigned its average bit score from the BLAST matches, plus the standard deviation. Support for differentiating between the artificial vector sequence and a background sample via differential cross-hybridization is enhanced when every artificial vector sequence's similarity to the signature set is higher than the background genomic sequence. It should be noted that the bit scores provide a rough estimate of hybridization potential and additional parameters may be used to optimize signature sets for a specific detection experiment and assay medium.

Two *k*-mer values, 30 and 60, were used with two signature set sizes, a smaller and larger set averaging 28,414 and 77,184 *k*-mers, respectively. Values for *k *(30 and 60) were chosen to examine signature types with different microarray hybridization patterns using lengths that we know from experience have different characteristics on our synthesized microarray platform. An alternative BLAST approach called MCS-only was included for comparison. MCS-only uses the multiple cloning sites of vectors exclusively as the source for creating signatures. The multiple cloning sites were first searched against the background sequence using BLAST, and regions without contiguous exact matches exceeding *k *were retained as input for constructing candidate signatures.

The MCS-only approach has the advantage of being easier to implement and requires less computational resources. Since the multiple cloning sites are expected to be good identifiers of vector sequence, it is possible that using all of the vector sequence as input provides limited information for creating signature data beyond what is already found at the multiple cloning sites. There are, however, potential disadvantages to this approach. Accessing the annotation specifying the multiple cloning site in every vector sequence is not easy. Despite our best efforts, we were unable to obtain multiple cloning site annotations for 18% of the completely sequenced vectors, although given the redundancy among vectors, the potential for extracting a good signature set is still possible.

**Figure 4:**
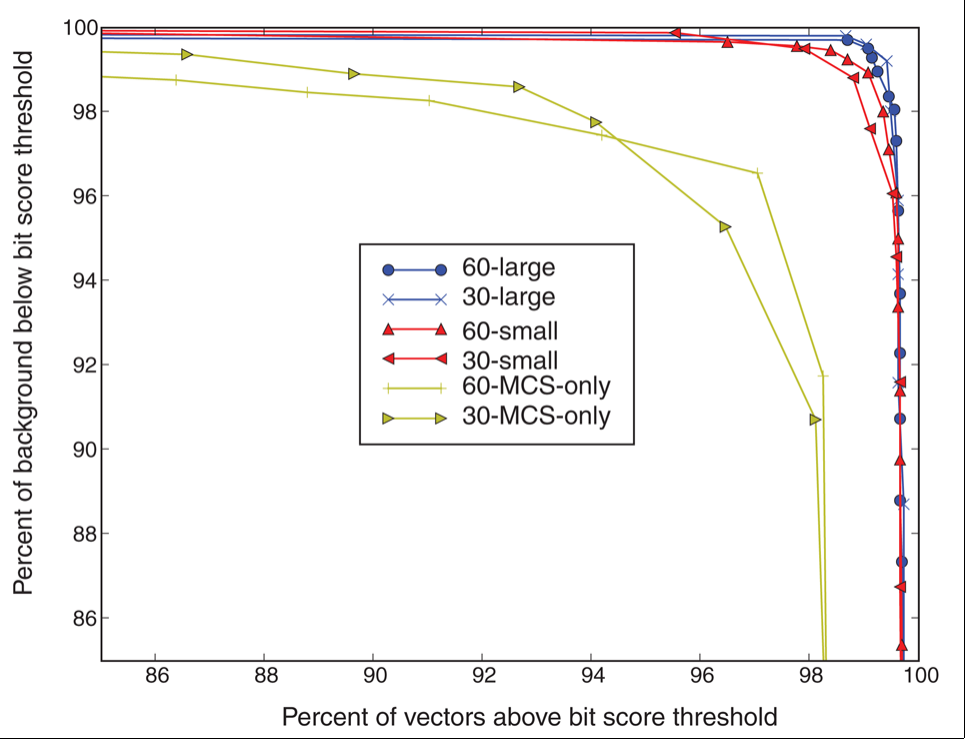
Artificial vector sequence detection. The percentage of correctly rejected background sequences (*y*-axis) versus correctly accepted artificial vector sequences (*x*-axis) using bit score thresholds. Each point is the percentage of background sequences (*y*-axis) with bit scores below a fixed bit score threshold versus the percentage of artificial vector sequences (*x*-axis) above the same bit score threshold. We examined 20 bit-score threshold values. Only the points with a rejection/acceptance percentage above 85% are shown. The six different signature sets are shown in the legend and are described by their *k*-mer size (30 and 60) and the signature set origin (large, small and MCS-only). The large and small sets are *k*-mer derived signature sets and MCS-only are signature sets derived exclusively from the multiple cloning site regions.

**Figure 5:**
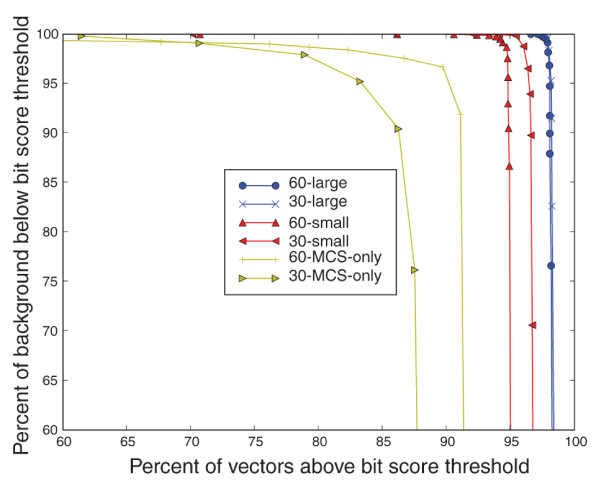
Artificial vector sequence detection with a modified signature set. The percentage of correctly rejected background sequences (*y*-axis) versus correctly accepted artificial vector sequences (*x*-axis) using bit score thresholds after filtering out signatures with high bit score matches to the background sequence.

Figure 4 shows the percentage of background sequences with bit scores below a given threshold (*y*-axis), versus the percentage of vector sequences with bit scores above the threshold (*x*-axis). Discrimination performance is slightly higher for the larger *k*-mer derived signature sets at most bit score thresholds. The MCS-only signature sets (30-MCS-only and 60-MCS-only in Figure 4) show substantially reduced performance compared with the more inclusive *k*-mer signature set approach. One key limitation is that the MCS-only signatures fail to correctly detect as many artificial vector sequences. The best MCS-only performance, 60-MCS-only, scored 98% of the artificial vector sequence above the background threshold but the threshold score had to be lowered to a level where only 92% of the background sequence would be rejected. The best *k*-mer derived signature set (60-large in Figure 4) by contrast scored 99% of the artificial vectors above the background threshold while rejecting 99.7% of the background sequence. Although the percentage of vectors detected and background sequence rejected is above 99%, a small percentage of background sequence still matched well with signatures. To reduce the potential for false positives, signatures with sequences similar to the background were removed. The resulting discrimination performance is shown in Figure 5. The *k*-mer derived signature sets show improved discrimination, with 100% of the background sequences scoring below a fixed threshold, while close to 98% of the vector sequence scored above the threshold. Thus, eliminating certain signatures reduced the potential for false positives while raising the percentage of missed vectors by only 1%. The best MCS-only signature set detection percentage (60-MCS-only in Figure 5) drops to 92% without raising the background sequence rejection percentage above 92%.

The results indicate that the limited annotation of multiple cloning sites for vector sequences is not the only cause for the drop in MCS-only performance. The signature-based approach yields additional signatures outside the MCS region that boost confidence in the prediction of a vector, particularly in cases where the MCS region does not match well with the signature set. An additional advantage of using signatures outside the MCS region is to recover more information about the detected vector. Since signatures can come from other functional regions such as replication of origin sites and selection marker genes, matches to these signatures could provide additional information that would be useful in learning more about a vector and host type embedded in a complex sample.

It is important to note that longer probe lengths reduce microarray hybridization specificity. Using shorter *k*-mer sizes for microarray probe design may lead to more specific detection rates compared with longer *k*-mers, since single nucleotide differences are used to determine candidate signatures for all values of *k*. The results in Figure 5 suggest that longer probes can be filtered using BLAST to remove additional near matches to the background, which could improve hybridization specificity while maintaining good coverage across the complete set of artificial vectors.

### Plasmid/vector conserved functional sequence

**Figure 6:**
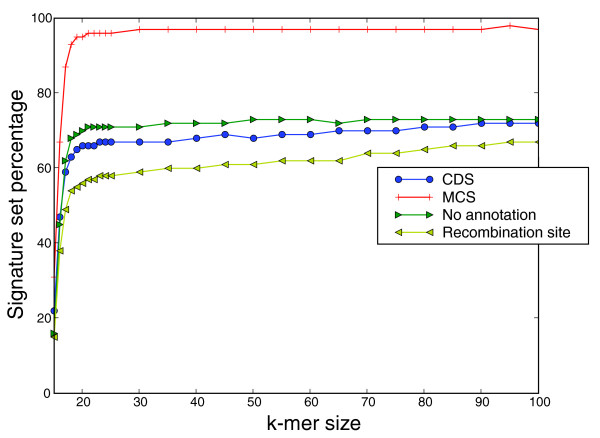
Signature set percentages for select functional annotation categories. Functional categories are protein coding genes (CDS), multiple cloning sites (MCS), no annotation and recombination sites.

Figure 6 shows the percentage of candidate signature sets for four select functional categories, coding sequence, multiple cloning sites, unannotated regions and recombination sites, for sets with at least 10 signatures and 10 *k*-mers. The highest percentage of signature sets are multiple cloning sites, confirming that these regions are a good source of signatures, followed by unannotated sequences. The functional category with the smallest percentage of signatures is the recombination site. As one might expect, Figure 6 shows that those regions subject to less-selective pressure yield higher numbers of candidate signatures; however, individual functional categories yield over 60% of the signatures (CDS in Figure 6). Although multiple cloning sites are an obvious choice for signature selection, in addition to limitations in access to functional annotation, continued development of recombineering methods [21], which use homologous recombination over restriction enzymes, mean that signatures from a range of functions should be included.

**Figure 7:**
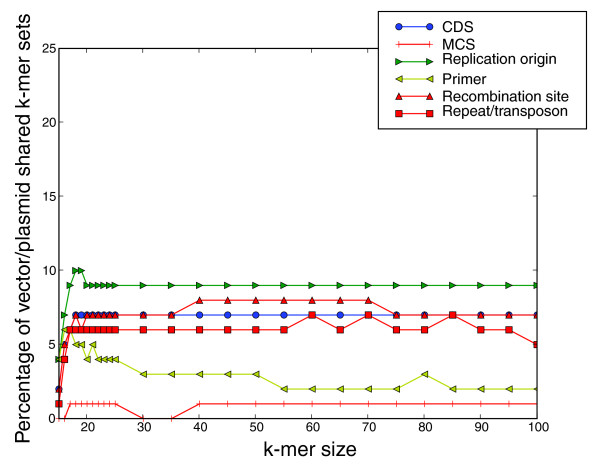
Vector/plasmid shared *k*-mer sets for select functional annotation categories. Percentage of shared *k*-mer sets is shown for different *k*-mer sizes.

Figure 7 shows the percentage of *k*-mer sets shared between vectors and natural plasmids but not with chromosomal sequences, organized by functional category. Understanding this distinction is important in determining where signatures may confuse natural plasmids with artificial vector sequences. Only 2.5 times as many *k*-mer sets are shared exclusively with the chromosomal data for *k *= 23 compared with sets shared exclusively with the natural plasmids, despite there being roughly 60 times as much chromosomal data. The origin of replication regions were found to be the most common functional category shared exclusively among natural plasmid and vector sequences while the multiple cloning sites and primer sites are very rarely vector/plasmid specific. Multiple cloning sites elements are most frequently specific to the artificial vector sequence, but in cases when they are not, they are found both in natural plasmids and chromosomes.

With the availability of interactive software tools for vector design [22], an automated procedure was developed to check for additional signature candidates in natural plasmids. Plasmids were searched against the *k*-mer sets to find cases where the sequence similarity to artificial vector sequence could support attempts to convert natural plasmids to novel vectors [23-26]. Including signatures with variations on the existing vectors could serve to deter attempts to evade detection using natural plasmids with small variations to known sequenced vectors. The 20-mers for each natural plasmid were mapped to the respective vector derived 20-mer sets; if the natural plasmid contained 90% or more of the 20-mers in a set, the natural plasmid was matched to the *k*-mer set. We found 21 natural plasmids from 10 bacteria and 5 non-species-specific plasmids with at least 3,000 *k*-mers in at least three annotated functional categories: coding sequence, replication origin and promoter, where *k*-mer sets have at least 50 *k*-mers. Table 1 lists the species names. Along with *E. coli*, other potentially hazardous bacteria are present such as the recently sequenced *Yersinia pestis biovar Orientalis str. IP275 *plasmid [27]. Any one natural plasmid shared *k*-mer set can be shared by tens or hundreds of vectors so vectors with the largest common number of *k*-mer sets were found to compare with previously used vectors, which could potentially support the use of a new vector [28].

**Table 1:** Bacteria with plasmids matched to artificial vectors.

*Enterococcus faecalis*	
*Escherichia coli*	
*Klebsiella pneumoniae*	
*Photobacterium damselae subsp. Piscicida*	
Environmental samples uncultured bacterium	
*Pseudomonas aeruginosa*	
*Salmonella enterica subsp. enterica serovar Typhi str. CT18*	
*Salmonella typhimurium*	
*Serratia marcescens*	
*Staphylococcus aureus*	
*Yersinia pestis biovar Orientalis str. IP275*	

Species with at least 3,000 20-mer matches to the vectors in three functional categories: protein coding gene, origin of replication region and promoter.

*Y. pestis *conserved 20-mer sets cluster into four distinct bacterial vector sets shown in Table 2. Each cluster specifies a common vector (or vectors). For example, the largest cluster labeled 1 in Table 2 contains kanamycin and streptomycin drug-resistant genes along with recombination and transcription termination sites, all mapping to two sequenced vectors (accession numbers [GenBank:4262403, GenBank:4323404]). Table 3 describes vectors for the clusters in Table 2. The common functional sequence between vectors and newly sequenced natural plasmids suggests inclusion of a supplemental set of natural plasmid-based signatures in genetic engineering detection assays.

**Table 2:** GenBank identifiers for vector sequence matching *Y. pestis* plasmid.

Cluster	*k*-mer sets	Vector GenBank accession	Functional elements
1	16	4262403, 4323404	Recombination site, CDS, promoter, transcription terminator
2	11	116119370, 984913	CDS, promoter, repeat region
3	2	120573441	Transcription terminator
4	2	Eight matching vectors	Origin of Replication

Columns list a numeric identifier (Cluster), the number of *k*-mer sets in the cluster, GenBank identifiers (when two or less vectors match), and the conserved functional elements.

**Table 3:** Summary description from the GenBank annotation of vectors matched to the *Y. pestis* plasmid.

GenBank accession	Host	Purpose	Comments
4262403	Broad range	Cloning	Gene cloning vectors for *Rhodobacter sphaeroides*
4323404	Broad range	Cloning	Gene cloning vectors for *Rhodobacter sphaeroides*
116119370	Unspecified	Cloning	The complete sequence of the BAC vector pECSBAC4
4984913	*Escherichia coli*	Cloning	Improved antibiotic-resistance gene cassettes and omega elements
120573441	Broad range	Expression	Analysis of transformation in *Acinetobacter baylyi*

Describes the vectors listed in Table 2.

## Conclusion

Candidate DNA signatures were found for nearly all artificial vector sequence. In a small number of cases overlap between natural plasmids and artificial vectors preclude detection with DNA signatures. With two exceptions, where the signatures were found at *k *= 23 and 47, the lack of signature coverage for a vector sequence was explained by the occurrence of an equivalent natural analog, which makes clear the limits of many vector/plasmid distinctions. Natural analogs must be included in vector based signature detection systems along with other natural plasmid derivatives, which could be used to evade detection from the existing core signature set. With the potential for plasmids to be converted into artificial vector sequence [29,30], developing predictive DNA signatures is an important challenge. At a minimum, signatures from the 21 plasmids sharing multiple functional elements with existing artificial vector sequence should be included to track potentially modified natural plasmids. Finding that 364 signatures cover nearly the complete set of vector sequences means that there is high sequence redundancy, making it feasible to maintain an expanding database of DNA signatures to track all sequenced vectors.

Future work should be directed towards bioassay design using DNA signatures on microarrays to test the efficacy of detecting genetically modified bacteria from a sample, which includes both modified and naturally occurring bacteria. We plan to collaborate more closely with scientists in the genetic engineering field to refine our bioinformatics tools to anticipate future natural plasmid-derived vector construction. As with any attempt to counter malicious use of technology, detecting genetic engineering in microbes will be an immense challenge that requires many different tools and continual effort. Cooperating with the scientific community to sequence and track available vector sequence will provide an opportunity for DNA signatures to support detection and deterrence against malicious genetic engineering applications.

## Materials and methods

Natural plasmid sequence was extracted from an Entrez query of taxonomic classification 'other sequence; plasmids' [31], GenBank plasmids and the Plasmid Database [32]. Sequences were checked for redundancy yielding the final natural plasmid sequence total of 65,341,821 bases in 1,567 contigs. In the pre-processed form there is overlap between the artificial vector set and the natural plasmid set. While some plasmids are naturally occurring, they are also used in genetic engineering. In cases where an engineered application is found, the sequence was treated as an 'artificial vector sequence'. The remaining artificial vector sequence was downloaded from the GenBank vector set available via anonymous ftp [33], ATCC [34], Virmatics [35] and an Entrez-based query of sequences classified taxonomically as artificial vector sequence. Vector sequences with fasta headers specifying eukaryote cell targets were removed, along with duplicate sequences. The background chromosomal sequence comes from the KPATH [9] database, which contains all available draft and completely sequenced microbial genomes (45,749 sequences totaling 4,057,440,823 bases).

### Signature pipeline

**Figure 8:**
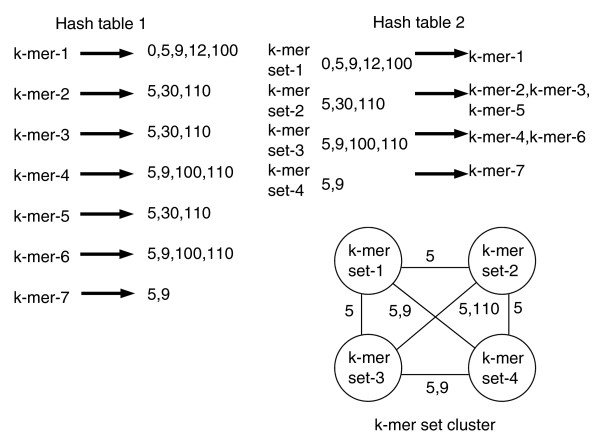
Hash tables and *k*-mer set clusters. The left panel shows schematic of an example hash table (Hash table 1). Each key is a *k*-mer (*k*-mer-1, *k*-mer-2,..., *k*-mer-7) with an entry storing a list of numeric identifiers for the sequences with the *k*-mer substring. The upper right panel shows the second hash table (Hash table 2), where each key is the set of *k*-mers common among the set of vectors specified by the key. The bottom right panel shows the graph representation of the four *k*-mer sets (numbered 1 to 4) with *k*-mer sets as nodes and labeled edges between nodes representing shared vectors between nodes.

Each vector sequence was assigned a unique integer identifier starting from 0 to the total number of sequences minus 1. A hash table was built with a hash key entry for each *k*-mer in the vector sequence and the numeric identifiers stored in order from the contributing sequences. An example schematic of the hash table is labeled 'Hash table 1' in Figure 8. As an example, the top entry in Figure 8 is *k*-mer-1 and is found in five different sequences: 0, 5, 9, 12 and 100. The computational cost to build the hash table is the number of *k*-mers (proportional to the total number of bases given as input) times the cost of inserting a pointer to the originating sequence for each *k*-mer into a sorted list, which is *O*(log *s*), where *s *is the total number of sequences and reduces to a constant value. This gives a linear runtime with respect to the number of nucleotides given as input. If the total number of input bases is *n *then there are *O*(*n*) bytes used for the keys times 2 * *s *bytes (assuming 2 bytes per integer). In theory, up to 3 TB of memory could be required, however, most *k*-mers are found in a smaller subset of sequences, dramatically reducing memory requirements. This problem can be viewed in the context of other multiple whole genome exact seed match comparison approaches that are potentially more memory efficient using variants of suffix trees [36,37] minus a step for chaining together conserved order blocks [16]. The principal difference is the need for a sequence clustering step, since *k*-mers are found in different subsets of the total set of input sequences.

Once the initial hash table is built, the sequence pointers of each *k*-mer entry become the keys for a second hash table, which records every combination of vector sequence with shared *k*-mers. A schematic of the hash table is labeled 'Hash table 2' in Figure 8. As an example, the second key from the top in Hash table 2 in Figure 8 forms a *k*-mer set called *k*-mer set-2, which shows that three sequences, 5, 30 and 110, share three *k*-mers, *k*-mer-2, *k*-mer-3 and *k*-mer-5. This comparative sequence approach presents a linear runtime with respect to the number of input nucleotides but has a theoretically high memory cost (owing to an exponential number of possible cluster combinations). In practice the entire study required less than 3 GB in online random access memory (RAM). Google sparse hash tables [38] were used to limit RAM consumption. DNA signatures are found by checking each nucleotide in the background dataset (natural plasmids and chromosomal sequence) and storing the *k*-mers shared with the initial vector derived hash table.

The background and vector sequences were searched against the signature set so that comparable sized query database sizes were used in the comparison. The background genomic sequence was searched against all five signature sets and the average result was taken. Default parameter values were used for BLAST. The second plot (Figure 5) shows signatures removed from the detection set using a bit score threshold of 100 and 50 for *k *= 60 and 30, respectively. A signature was removed if it has at least one match with bit score above the threshold. The two *k*-mer based signature set sizes were chosen from two different criteria. The larger set was taken by selecting the first 10 signatures from each *k*-mer set (chosen at random). The smaller set was chosen by taking a maximum of the first 10 signatures per vector sequence selecting signatures shared by the largest number of vectors.

### Matching vectors with plasmids

**Figure 9:**
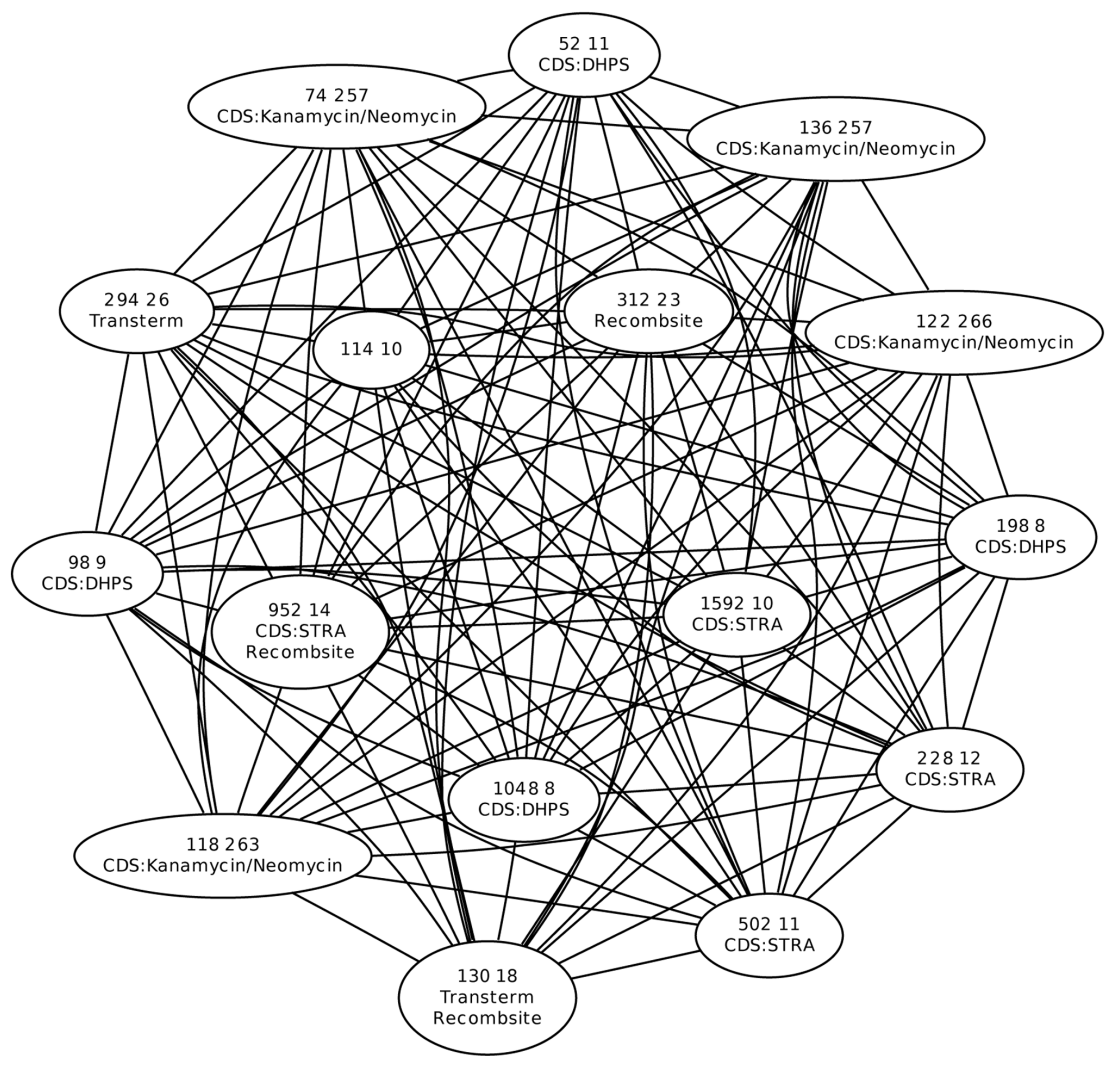
*k*-mer set cluster. Graph of cluster 1 from Table 2. Each node shows the number of *k*-mers in the set (left number), the number of artificial vectors sharing the *k*-mer substrings (right number) and the functional annotation. Edges denote common vectors between two nodes. Abbreviations are as follows: DHPS, dihydropteroate synthase; STRA, streptomycin resistance; Kanamycin/Neomycin, Kanamycin/Neomycin resistance; Recombsite, recombination site; Transterm, transcription termination.

The vector sequences with the greatest number of *k*-mer sets shared with a natural plasmid of interest (such as the *Y. pestis *plasmid given as an example) were found using a graph theoretic approach. Each *k*-mer set is a node in a graph, with labeled edges between two nodes listing the vectors in common. An example is shown in Figure 8. In general, if *k*-mer set *A *and *k*-mer set *B *are two nodes in the graph, node *A *contains the *k*-mers shared by the vectors $V_{A}=v_{1}^{A},v_{2}^{A},...,v_{x}^{A}$ and node *B *contains the *k*-mers shared by the vectors $V_{B}=v_{1}^{B},v_{2}^{B},...,v_{y}^{B}$. An edge between *A *and *B *exists if the intersection between *V*_*A *_and *V*_*B *_is non-empty and the edge is labeled with the names of the shared vectors. For example, in Figure 8 there is an edge between *k*-mer set 1 and *k*-mer set 4 labeled with their common vectors 5 and 9. Finding the *k*-mer sets with the greatest number of common vectors finds the maximal clique in the graph [39] with the added constraint that every edge in the clique must share at least one vector in common with every other edge in the clique. Once the maximal clique with edge label constraints is found, it is removed from the graph and the process is repeated for the remaining *k*-mer sets until all nodes (*k*-mer sets) are assigned to a maximal clique. The cluster labeled 1 in Table 2 is shown in graph form in Figure 9; for clarity the edge labels are not shown, but each edge is labeled with the two common matching vectors: [GenBank:4262403] and [GenBank:4323404].

## List of abbreviations

CDS, coding sequence; MCS, multiple cloning site; PCR, polymerase chain reaction, RAM, random access memory.

## Authors' contributions

JEA, SNG and TRS conceived and designed experiments. JEA implemented experiments and drafted the manuscript. All authors read and approved the final manuscript.

## Additional data files

The following additional data are available with the online version of this paper. Additional data file 1 is the list of artificial vector identifiers. Additional data file 2 is the list of natural plasmid identifiers. Additional data file 3 is the complete set of 30-mer signatures used in the cross-validation set. Additional data file 4 is the complete set of 60-mer signatures used in the cross-validation set.

## Supplementary Material

Additional data file 1 The list of artificial vector identifiers. Click here for additional data file

Additional data file 2 The list of natural plasmid identifiers Click here for additional data file

Additional data file 3 The complete set of 30-mer signatures used in the cross-validation set Click here for additional data file

Additional data file 4 The complete set of 60-mer signatures used in the cross-validation set Click here for additional data file
